# Towards Tabula Gallus

**DOI:** 10.3390/ijms23020613

**Published:** 2022-01-06

**Authors:** Masahito Yamagata

**Affiliations:** Department of Molecular and Cellular Biology, Harvard University, Cambridge, MA 02138, USA; yamagatm@mcb.harvard.edu or yamagatm2@gmail.com

**Keywords:** chicken, *Gallus gallus*, birds, single-cell RNA sequencing, transcriptome, cell atlas, embryology, bioimaging, antibodies, evolution

## Abstract

The Tabula Gallus is a proposed project that aims to create a map of every cell type in the chicken body and chick embryos. Chickens (*Gallus gallus*) are one of the most recognized model animals that recapitulate the development and physiology of mammals. The Tabula Gallus will generate a compendium of single-cell transcriptome data from *Gallus gallus*, characterize each cell type, and provide tools for the study of the biology of this species, similar to other ongoing cell atlas projects (Tabula Muris and Tabula Sapiens/Human Cell Atlas for mice and humans, respectively). The Tabula Gallus will potentially become an international collaboration between many researchers. This project will be useful for the basic scientific study of *Gallus gallus* and other birds (e.g., cell biology, molecular biology, developmental biology, neuroscience, physiology, oncology, virology, behavior, ecology, and evolution). It will eventually be beneficial for a better understanding of human health and diseases.

## 1. Introduction

The chicken (*Gallus gallus*) is the most common domesticated animal that originated from the red jungle fowl in Southwest Asia [1,2]. Chickens are not only consumed widely for their eggs and meat, but are also used for feathers, ornamental pets, crowning, and fighting in some locales. Chickens are also crucial in the production of vaccines by growing viral particles in the allantoic fluid of live eggs. However, most importantly, chickens are one of the most valuable model animals for biological and medical research [3,4]. The purpose of this review article is to advocate the importance of the basic science of chickens and propose a “Tabula Gallus” (tabula: tablet or map), a cell atlas that integrates a gamut of multimodal information including single-cell RNA sequencing (scRNA-seq) data.

Basic knowledge of chickens is essential for avian biology in general. Furthermore, other laboratory species, including pigeon, quail, duck, zebra finch, ostrich, emu, owl, crow, and parrot, have also been exploited as animals to better understand their biochemistry, physiology, anatomy, and behavior such as bird song and intelligence. In addition, numerous wildlife species, including migratory birds, have contributed to ecological and ornithological studies. Although other avian species can be selected as a decent model to study specific questions [5], it is widely accepted that chickens are the best bird model because of their accessibility: chickens and eggs can be readily obtained in large numbers throughout the year and in many locations. 

## 2. Background

### 2.1. Pasteur, Darwin, and Cajal

Readers may recall that several major biological concepts for the understanding of *Homo sapiens* were initiated by looking at birds and their body parts. In the late 19th century, Louis Pasteur (1822–1895) accidentally discovered the first attenuated vaccine for chicken cholera, which subsequently inspired him and others to vaccinate for other infectious diseases [6]. At a similar time, mockingbirds and finches in the Galápagos islands played a critical part in the conception of the theory of evolution by Charles Darwin (1809–1882) [7]. Around the time between the 19th and 20th centuries, Santiago Ramón y Cajal (1852–1934) extensively used avian brains and retinae for his Golgi preparations and formulated the neuron theory [8]. More recently, Konrad Lorentz (1903–1989) established neuroethology by discovering imprinting of a young goose, underscoring potential extrapolation from animal behavior to humans [9]. 

Several landmark discoveries in biochemistry and molecular biology were also made using birds. Just before the 20th century, Christiaan Eijkman (1858–1930) noticed that the symptoms of beriberi in chickens were recovered when the birds were fed with unpolished rice, leading to the discovery of the anti-beriberi factor (now called ‘vitamin B1’) [10]. Szent-Györgyi Albert (1893–1986) studied cellular respiration using minced bird muscle and identified fumaric acid and other steps, which are now known as the TCA cycle [11]. Peyton Rous (1879–1970) discovered a transmissible retrovirus, now acknowledged as the Rous sarcoma virus, from a chicken sarcoma [12]. The research on this virus later led to the discovery of the reverse transcriptase [13], and the *src* oncogene in normal cells [14]. Rita Levi-Montalcini (1909–2012), working in the Viktor Hamburger (1900–2001) laboratory at Washington University in Saint Louis, grafted an aggregate of mouse sarcoma cells to developing chick embryos and discovered that the tumor secreted a factor that stimulated the growth of nearby sensory and sympathetic ganglia [15]. Her collaborator Stanley Cohen (1942–2013) isolated the factor, later called nerve growth factor (NGF), which is considered the earliest growth factor discovered [16]. These paradigm-shifting concepts, including the neuron doctrine, imprinting, vitamins, the TCA cycle, oncoviruses, and growth factor, won the Nobel Prize in Physiology or Medicine during the 20th century. 

As evidently exemplified by NGF, the most important discipline influenced by chicken and chick embryos is perhaps the field of developmental biology [3,17,18]. In ancient Greece (ca. 330 BCE), Aristotle recorded the first observation on developing chick embryos [19]. At the dawn of modern science, William Harvey (1578–1657) and Marcello Malpighi (1628–1694) observed chick embryos and studied the anatomy and development of blood vessels [20]. At the end of the 19th century, Entwicklungsmechanik, advocated by Wilhelm Roux (1850–1924), promoted the use of chick embryos [21,22]. C. H. Waddington (1905–1975) also used chick embryos and analyzed the mechanism by which the embryonic axis and left–right asymmetry can be established [23]. 

Subsequently, using chick embryos has profoundly influenced developmental biology since the middle of the 20th century [3,24], not only for understanding the fundamental processes in development, but also in the function of modeling human development and disorders, as previously summarized in some landmark papers and comprehensive reviews [25,26,27,28,29,30,31,32,33,34,35,36,37,38,39,40,41,42,43,44,45,46,47,48]. 

### 2.2. Many Advantages

Like mammals, birds breathe air and are endothermic animals, offering advantages compared to other ectothermic models. For example, enzymes, binders, and structural proteins are adapted for warm temperatures. Avian bodies and cells also provide platforms where the activities of xenotypic proteins and synthetic drugs can be examined in vivo and in vitro. A variety of dissociated cells and explants from chick embryos can be maintained cultured to address important cell biological issues [49,50,51,52,53,54]. It is also useful to generate chimeras by transplanting cells and tissues from other endothermic animals (e.g., chick-quail chimera) [28]. 

It is often overlooked that chicks, chick embryos, and eggs are scalable sources for extracting and isolating bioactive materials. For example, some functional proteins were purified biochemically from thousands of chick embryos and characterized (e.g., references [55,56,57]). Moreover, it should be worth mentioning that the sequences of chicken proteins are likely different from those of mammalian proteins, being favorably antigenic to mammals (see below). Egg yolk immunoglobulins (IgY), the chicken equivalent of mammalian IgG, are transferred to egg yolk and readily purified for various applications [58]. 

Nonetheless, in 2004, Claudio Stern noted that “in more recent days there have been signs of ‘anti-chick’ racism by institutions hiring new young faculty, reviewers of grant applications and even some reviewers of submitted manuscripts.” [59]. This scathing view might still be valid. In general, we need to broaden our range of model animals to include more species, as Jessica Bolker recently discussed [60,61]. Fortunately, it is now empirically recognized that chickens are one of the leading model animals, because they can recapitulate human development and physiology well [62].

The culmination of decades of progress in chicken biology is that the genome sequencing of chicken was completed in 2004 [63]. The relatively compact genome size (~1.1 Gb) makes the species ideal for whole genome-based gene association and gene expression-based analyses [64]. In addition, an extensive effort to sequence avian genomes B10K is currently underway [65,66]. 

Recent advancements in germline and somatic transgenesis have allowed further understanding of genes, molecules, cell types, and their relationships in detail. For example, electroporation for the study of the effect of adding or silencing a gene has been used for various purposes [67]. Other tools for studying and perturbing their genetic makeup are chicken embryonic stem cells and primordial germ cells [68,69,70,71], piggyBac and Tol2 transposons [72,73,74], and various viral vectors [71,75,76,77,78,79,80]. More recently, CRISPR-based genome editing technologies have allowed us to precisely link the expression of a single gene directly to cells expressing the gene by inserting reporter genes to the specific chromosomal locus in birds [81,82,83,84]. Combined with these molecular techniques, breakthroughs in bioimaging such as super-resolution microscopy and spatial transcriptomics will potentially provide a deep insight into the complicated spatiotemporal changes during development [85,86]. 

Remarkable progress in single-cell profiling technologies has revolutionized our ability to study multicellular organisms at an unprecedented resolution [87]. Thus, leveraging these proof-of-concept advances to build a comprehensive atlas of *Gallus gallus* at the cellular resolution is an ambitious and timely endeavor similar in scale to the genome project.

## 3. Tabula Gallus

### 3.1. Chicken Cell Atlas

**Figure 1:**
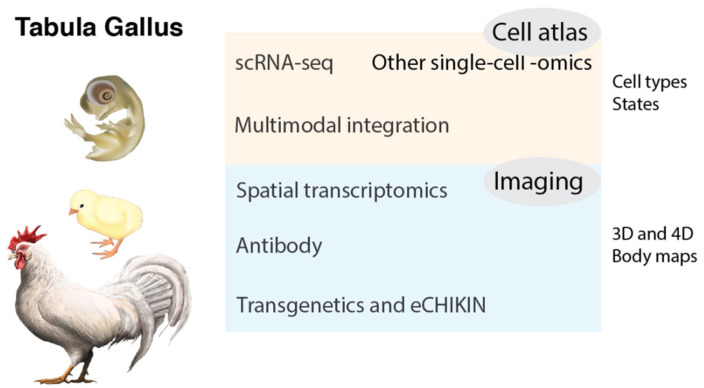
Tabula Gallus will generate a compendium of single-cell transcriptome data from *Gallus gallus*, characterize each cell type, create 3D and 4D body maps, and provide tools for the study of the biology of this species. Images: ©2016 DBCLS TogoTV/CC-BY-4.0.

Tabula Gallus will generate a compendium of single-cell transcriptome data from *Gallus gallus*, characterize each cell type, and provide the tools for studying the biology of this species, like other ongoing cell atlas projects (Tabula Muris and Tabula Sapiens/Human Cell Atlas for mice and human, respectively) [88,89,90,91] (https://tabula-muris.ds.czbiohub.org (accessed on 10 December 2021); https://www.humancellatlas.org (accessed on 10 December 2021); https://tabula-sapiens-portal.ds.czbiohub.org (accessed on 10 December 2021)). Tabula Gallus will potentially become an international collaboration among many researchers. This project will be useful to understand the basic sciences of *Gallus gallus* and other birds (e.g., cell biology, molecular biology, developmental biology, neuroscience, physiology, oncology, virology, behavior, ecology, and evolution). In addition, it will eventually be beneficial to the knowledge of human health and diseases. Here, the overall picture of Tabula Gallus is summarized (Figure 1). 

### 3.2. Cell Types and States

A cell is a basic unit of architecture and function in biological organisms, and animals have evolved an immense variety of distinct cell types with specialized roles. Although cell types have initially been identified on the basis of morphology and phenotype, the application of molecular methods has enabled a more precise characterization of each cell type. scRNA-seq technologies allow the detection of gene expression at single-cell resolution, which has been revolutionizing transcriptomic analyses [87,92].

scRNA-seq dissects cell types and states and provides more information at a higher granularity by profiling massive numbers of cells. There are a few advantages of chickens for single-cell technologies. Because of their relatively large size (e.g., compared with *Drosophila melanogaster* (fruit fly) and *Dario rerio* (zebra fish)), dissecting organs and tissues from whole animals is relatively straightforward. It is also practical to use numerous eggs and animals to harvest many cells. Thus, it is powerful to profile more cells at an affordable cost, capturing rare cell types and nuanced states during embryonic stages. For humans and rare animals, single-nucleus RNA sequencing (snRNA-seq), instead of scRNA-seq, is standard due to the availability of fresh tissues [93]. Although snRNA-seq has advantages over scRNA-seq in some cases, it is helpful to use both techniques for chickens. 

One precedent is the Tabula Muris project which created a compendium of single-cell transcriptome data from the experimental model organism *Mus musculus* comprising nearly 100,000 cells from 20 organs and tissues [88]. The data from this project will uncover many novel aspects of cell biology, including gene expression in barely characterized cell populations and the characterization of new types of cells in many tissues.

The Tabula Gallus is currently at an early stage (~20 scRNA-seq studies using chicks as of early 2022) [89,90,91,92,93,94,95,96,97,98,99,100,101,102,103,104,105,106,107,108]. Nonetheless, a couple of fledging studies support the feasibility of this project revealing diverse cell types and their states in the chick limb buds [96,102], the early primitive streak stage [95,103], neural crests [94,100], and the developing neural retina [98,107]. Strikingly, in the developing neural retina, 136 cell types plus 14 positional or developmental intermediates were identified, providing a foundation for studying diverse neuronal cell types in the central nervous system [107], supporting the unique function of the avian visual system [109]. In addition, scRNA-seq of retinal Müller glial cells revealed genes potentially controlling retinal regeneration [99] as well as positional and developmental states [107]. 

The raw data and references of many single-cell studies are searchable at NCBI’s GEO (https://www.ncbi.nlm.nih.gov/geo/ (accessed on 10 December 2021)) and some single-cell reference sites (e.g., https://panglaodb.se/papers.html, https://www.nxn.se/single-cell-studies/ (accessed on 10 December 2021)), respectively. To explore the large and complex datasets, the single-cell data are generally encouraged to deposit to the interactive viewers for single-cell data (Single Cell Portal, https://singlecell.broadinstitute.org; (accessed on 10 December 2021) UCSC Cell Browser, https://cells.ucsc.edu (accessed on 10 December 2021); EMBL-EBI, https://www.ebi.ac.uk/gxa/sc/home (accessed on 10 December 2021)). However, those datasets are separated. Thus, the first step towards “Tabula Gallus” would be to create an integrated website to catalog those datasets and facilitate international collaboration between many researchers. The precedents would be Tabula Muris and Tabula Sapiens/Human Cell Atlas [88,89,90,91]. Moreover, to combine further information such as validated gene expression data and images (see below), the species-specific community websites like GEISHA (http://geisha.arizona.edu (accessed on 10 December 2021)) may provide excellent starting points as a resource for chicken biology as in the cases for other model animals (e.g., https://flybase.org (accessed on 10 December 2021), https://wormbase.org/ (accessed on 10 December 2021)). 

### 3.3. More Modalities: Epigenome, Protein, Glycan, and Connectome

Recent single-cell molecular profiling based on transcriptome, accessible chromatin sequencing, DNA methylome, or selective protein expression patterns have enabled a subtler distinction of cell types [87,110,111]. These multiple modalities further facilitate the resolution of cell types and states and provide more information. 

The integration of epigenome features with diverse omics datasets facilitates multimodal omics analyses, providing a chance to explore various biological events from novel perspectives. For example, a series of single-cell sequencing methods for the detection of heritable DNA base modification by methylation and chemical and structural modifications of chromatin allow the unraveling of epigenetic changes on a genome-wide scale. In particular, a single-cell sequencing assay for transposase-accessible chromatin (scATAC-seq) is the most widely used technique for the study of epigenetic landscapes in single cells [89,112]. Dynamic changes in the epigenome play pivotal roles in normal development during organogenesis and homeostasis and in diseases. 

Protein expression signatures also help to define cell types and states. Transcriptomes are often used to represent protein expression, but it has long been noticed that the abundance of mRNA and proteins is not correlated because of the complex post-transcriptional/translational regulation and protein degradation. Some of the latest approaches, such as CITE-seq (Cellular Indexing of Transcriptomes and Epitopes by Sequencing), REAP-seq (RNA Expression and Protein Sequencing assay), and Ab-seq, complement single-cell transcriptomes with concurrent protein assessments by marking dissociated cells with oligo-tagged immunoglobulin antibodies [113,114,115,116]. SUGAR-seq instead employs oligonucleotide-labeled lectins to analyze the glycoconjugates and RNA simultaneously [117]. Thus, these approaches can be used to determine the quantitative correlation between gene expression and other modalities at the single-cell level. 

Besides these molecular modalities, the spatial information and morphology of cells are also useful factors to evaluate cell types and states (see below). For neurons, other modalities such as electrophysiology and connectome are critical for classifying cell types and assessing the transition [86,118,119,120,121].

### 3.4. Temporal and Spatial Transcriptomics

The cell types are generally categorized and defined by existing information from common organisms and adult cellular profiles, which may not accurately reflect embryonic or transient cell types present only during development and intermediate steps of differentiation. To address this issue, it is vital to validate the expression of crucial marker genes in each tentative cell type. One traditional way would be to carry out in situ hybridization using a synthetic RNA probe or RNAScope™ assay. These experiments can be typically performed on tissue sections or occasionally in whole mounts. Moreover, many time points need to be observed to improve assessment, and transient cell states often need to be mapped back into their three-dimensional space in vivo. Furthermore, it is also helpful to observe the expression of multiple genes simultaneously on the same tissue sections and cells to reveal heterogeneous cell populations. For example, state-of-the-art imaging methods such as multiplexed error-robust fluorescence in situ hybridization (MERFISH), Seq-FISH, and CARTANA™ technologies allow the measurement of the copy number and spatial distribution of numerous transcripts in single cells and sections at the same time [86,120,121,122]. 

In parallel, the application of high-throughput genomic technologies to tissue sections in situ is beginning to provide data of striking resolution. For example, in Slide-seq and Visium™ technologies, RNA is transferred from freshly frozen tissue sections onto a monolayer of DNA-barcoded beads and a glass slide with barcoded spots with known positions, respectively [123]. Data from such spatial transcriptomics from tissue sections can integrate with scRNA-seq profiles from dissociated cells, facilitating the interpretation of cell types and states based on scRNA-seq and spatial transcriptomics [87]. 

Tabula Gallus will be able to borrow various standard methods that have been used in ongoing prolific cell atlas projects on *Homo sapiens*, *Mus musculus*, and their embryos. For example, in such cell atlas projects, a standard coordinate system for locations in the animal body (a common coordinate framework (CCF)) is crucial [124,125].

### 3.5. Antibodies and Bioimaging

Antibodies, especially for use in immunohistochemistry, represent one of the most powerful tools in understanding the localization and function of the gene products and the cells that express them. It is not usually challenging to raise antibodies to avian proteins in hyperimmunized mammals (e.g., mice and rabbits), albeit this is not commonly carried out. This is because most, if not all, bird-derived protein sequences are divergent from their mammalian counterparts: chicken proteins are likely antigenic to mice and rabbits when injected. Each antigen can be typically obtained as a bacterially expressed fusion protein or synthesized peptide. It is also possible to apply a series of molecular display platforms such as phage and microbial surface display and nucleic acid immunization [126,127,128]. The mammalian cell display procedure has successfully generated a series of murine antibodies to chick proteins [107]. The Developmental Studies Hybridoma Bank offers many monoclonal antibodies for developmental studies of chickens (https://dshb.biology.uiowa.edu/ (accessed on 10 December 2021)).

Combined with whole-mount immunostaining or in situ hybridization, still-improving tissue clearing methods to render organs and tissues transparent can provide molecular profilings at a cellular or subcellular resolution and help to create 3D and 4D body expression maps [129]. In addition, applying artificial intelligence and machine learning algorithms to address image analytical issues will also be used [130].

### 3.6. CRISPR-Mediated and Homology-Instructed Knock-In

To reveal the structure and functions of molecularly defined cell types as well as the biological function of genes, germline transgenesis is well established in the most commonly used animals (mice, zebrafish, *Drosophila*, and *C. elegans*). For example, transgenic animals can be generated in which regulatory elements from a gene specifically expressed by a cell type of interest regulate the expression of a reporter such as GFP [131]. Although this powerful strategy cannot apply to humans, various reporter lines have been developed to observe the morphology of cells. These reporter lines are created by introducing foreign DNA into the germ line or selectively knocking-in the DNA sequence to the germ line using homologous recombination.

Although germline transgenesis for chickens remains a challenging method, generating and maintaining transgenic chicken lines in special facilities are still feasible [132]. However, simpler methods were needed to specifically label somatic cells based on the genes that they express. eCHIKIN (electroporation- and *C*RISPR-mediated Homology-Instructed Knock-IN) is a method for CRISPR-based genome editing in somatic cells to insert GFP reporters or Cre recombinase into genes identified as cell-type selective in the scRNA-seq data [84]. This method involves in ovo electroporation of developing chick embryos. The initial study on the chick retina demonstrated that eCHIKIN is highly reproducible and can be used for many genes [107]. Such molecular genetical methods will correlate morphological and physiological data with single-cell technologies. Other genetic reporters such as APEX2 and HRP can also be used to further study the detailed structure of individual cells in electron microscopy [133].

### 3.7. Multimodal Integration and Computational Biology

As discussed above, Tabula Gallus will integrate all the modalities and generate a compendium of single-cell experimental data, including the transcriptome, epigenome, proteome, glycome, morphology, electrophysiology, and connectome. Experimental developmental biology has been based on ontogenic relationships of embryonic cells to defined adult cell types. I expect that the Tabula Gallus project will move towards in silico cell ontology that integrates multimodal single-cell data and empirical knowledge of embryonic cell types from chicken and other model animals. Computational approaches including big data and machine learning are promising because chick embryos have been used as a framework for “wet” developmental biology to address fundamental issues such as cell lineage, differentiation, cell migration, and cell interactions.

The most familiar analysis of single-cell data would be the determination of the lineage relationships among diverse cell types and states′ cell lineages that lead to the formation of tissues, organs, and complete organisms. Historically, the lineage relationships were analyzed by observing intact and manipulated embryos or cultured cells in vitro under microscopes [134]. Various heritable reporters have been subsequently used for lineage tracing in a retrospective manner [135,136]. However, such studies needed to introduce experimental reagents, such as replication-defective retroviral vectors, transposable elements, or recombinases. Using single-cell data, latent trajectory (pseudotime) analyses in silico have opened up new ways of estimating cellular transitions during various processes such as cell differentiation, cell cycle, and dynamic response to various stimuli [137,138,139]. Likewise, RNA velocity is an advanced bioinformatic method based on the changing rate of gene expression for a certain gene at a particular moment on the ratio of its spliced and unspliced mRNA, speculating cellular transitions [140].

In parallel, new techniques that simultaneously retrieve single-cell transcriptomic data and genetic lineage markers from the same cell have been developed [139]. For example, scGESTALT and ScarTrace combine CRISPR-Cas9-based scarring and single-cell transcriptomes to obtain both cell clonality and identity [141,142]. intMEMOIR allows single-cell analysis of cell lineage, transcriptome, and spatial information in the same tissue [143]. These advanced methods may have applications in chick embryos to understand how the mature cell atlas is built from the embryonic cell atlas at the single-cell level.

Bioinformatics can also be used to predict cell–cell interactions at the spatial layer. Cell–cell interactions mediated by ligand–receptor pairing are critical during development and the adult body. For example, CellPhoneDB, a repository of ligands, receptors, and their interactions, combines a statistical framework to predict cell–cell interactions between two cell types inferred from single-cell data [144].

### 3.8. Comparative Transcriptomics and Evolution of Cell Types

There is much advancement in generating single-cell transcriptomic and multimodal atlases to illustrate the cell types within individual species. Charles Darwin conjectured that evolution is like “Tree of Life” in his 1859 book On the Origin of Species [7]. Ever since, biologists have tried to draw trees of life. Powerful DNA sequencing technologies and phylogenic analyses revolutionized that project, finding the relationship among species encoded in their genes. In addition to organisms and their genes per se, their cells and organs also underwent evolution. The ultimate product of evolution would be a human brain, whereas primitive animals possess simple nervous systems. Accordingly, a repertoire of neuron types in the brain was also dramatically altered during evolution [145,146,147,148]. Like many neuron types, general cell types are evolutionary units that have diversified in structure and function since the beginning of multicellularity [149,150,151,152].

During normal development, cell types are established by gene regulatory networks comprised of regulatory molecules such as transcription factors and signaling pathways in the context of cell lineages and surrounding environments [153]. Likewise, cells are diversified in evolution by changing the networks, generating a series of novel cell types [149,154]. A plausible and parsimonious mechanism of cell-type creation is cell-type duplication, during which new cell types emerge by modifying developmental lineages, although the evolutionary and the developmental lineage of cell types are not necessarily the same. In parallel, some cell types are maintained by preserving the same regulatory networks over vast expanses of evolutionary time. Thus, the use of cross-species cell type comparisons of scRNA-seq data promises to address questions regarding the evolutionary origin of cell types and species-specific cell types. Such cell-type phylogenetics over very long evolutionary spans and phyla (mammals vs. aves vs. fish) has generally been considered challenging. However, recent progress shows that such analysis leads to being able to envision the function of novel cell types even in human tissues [107,147,150,151,152,155].

## 4. Beyond Tabula Gallus

Taken together, the cumulative resource from the Tabula Gallus will provide the foundation for a comprehensive collection of transcriptomic biology to facilitate the basic scientific study of *Gallus gallus* and other birds. The disciplines benefiting from this will include cell biology, molecular biology, developmental biology, neuroscience, physiology, oncology, virology, behavior, ecology, evolution, poultry/food industries, and medicine. For example, various cell culture methods, as well as simple egg incubation, may provide an opportunity to develop pharmacological assay systems by which to understand and screen drugs [38]. Finally, understanding the structure and function of avian brains at the cellular and molecular level could open a new avenue to unravel their logistics and genetic programs for their astonishing cognition and natural intelligence [109,156,157,158].
